# Exploring Postpartum Depression : Sociodemographic and Pregnancy-Related Influences

**DOI:** 10.1192/j.eurpsy.2025.1302

**Published:** 2025-08-26

**Authors:** N. Messedi, O. Bouattour, F. Khanfir, R. Ben Jmeaa, I. Chaari, F. Charfeddine, L. Aribi, K. Chaabene, J. Aloulou

**Affiliations:** 1 Psychiatry « B » department; 2 Gynecology department, Hedi Chaker University Hospital, Sfax, Tunisia

## Abstract

**Introduction:**

Postpartum depression is a widespread mental health condition affecting mothers in the weeks and months following childbirth, with potentially significant impacts on both maternal and child well-being. Several factors have been linked to an increased risk of postpartum depression, including perceived social support, sociodemographic characteristics, and pregnancy-related factors.

**Objectives:**

This study aims to investigate postpartum depression prevalence among postpartum women and its associated factors.

**Methods:**

We conducted a cross-sectional descriptive and analytical study targeting women in their first week postpartum who had been admitted to the gynaecology-obstetrics department of the Hedi Chaker University Hospital in Sfax, Tunisia. The study was conducted over a three-month period (October, November and December 2023). To assess the prevalence of postpartum depression, we used the Tunisian Arabic version of the Edinburgh Postnatal Depression Scale (EPDS). A cut-off score of 10 or more on the EPDS Scale indicates the presence of postpartum depression.

**Results:**

Our sample consisted of 220 paturients. The mean age of patients was 31.1 ± 6.6 years. Among the participants, 50.9% came from rural areas. Unwanted pregnancy was reported by 41.8%, with 13.2% having considered abortion. Socioeconomic challenges were notable, with 32.7% of participants reporting low socioeconomic status. Poor marital relationships were reported by 10.9% of the parturients. Additionally, 7.3% had a poor relationship with their mother.

Post partum depression (EPDS ≥ 10) was present in 20.9% of participants.

Depression was significantly linked to: younger age (p=0.001), being single or divorced (p=0.003), lower level of education (p=0.005), unemployment (p=0.03), and low socioeconomic status (p=0.007). Poor marital relationships also showed a strong association with depression (p=0.013). Additional risk factors included unwanted pregnancy (p=0.003), difficulty accepting the pregnancy (p<0.001), exaggerated sympathetic signs (p=0.011), lack of pregnancy follow-up (p=0.004), and pregnancy complications such as gestational diabetes and hypertension. Depression was also associated with post-partum stressors, including spontaneous labor (p=0.044), complications after delivery (p=0.022), infant malformations (p=0.001), prematurity (p=0.001), poor infant health (p<0.001), and lack of breastfeeding (p<0.001).

**Conclusions:**

These findings highlight the role of both socioeconomic and pregnancy-related factors in maternal mental health. Understanding these interconnected factors is essential for identifying at-risk populations and implementing targeted interventions to support maternal mental health.

**Disclosure of Interest:**

None Declared

